# Earlier Comprehensive Cancer Genomic Profiling in Gynecologic Cancers May Facilitate Genotype‐Matched Therapy: A Prospective Single‐Institution Study

**DOI:** 10.1155/ogi/9990671

**Published:** 2026-06-18

**Authors:** Kazusa Nofuji, Tasuku Mariya, Tomohiro Kubo, Ayako Murota, Mizue Teramoto, Shutaro Habata, Motoki Matsuura, Masahiro Iwasaki, Aki Ishikawa, Kohichi Takada, Masashi Idogawa, Ichiro Kinoshita, Akihiro Sakurai, Tsuyoshi Saito

**Affiliations:** ^1^ Department of Obstetrics and Gynecology, Sapporo Medical University School of Medicine, Sapporo, Japan, sapmed.ac.jp; ^2^ Division of Clinical Genomics, Department of Genomic and Preventive Medicine, Sapporo Medical University School of Medicine, Sapporo, Japan, sapmed.ac.jp; ^3^ Department of Medical Oncology, Sapporo Medical University School of Medicine, Sapporo, Japan, sapmed.ac.jp; ^4^ Department of Gastroenterology and Hepatology, Sapporo Medical University School of Medicine, Sapporo, Japan, sapmed.ac.jp; ^5^ Department of Obstetrics and Gynecology, NTT Medical Center Sapporo, Sapporo, Japan; ^6^ Iwasaki Women’s Health Clinic, Tomakomai, Japan; ^7^ Division of Medical Genome Sciences, Department of Genomic and Preventive Medicine, Sapporo Medical University School of Medicine, Sapporo, Japan, sapmed.ac.jp; ^8^ Division of Clinical Cancer Genomics, Hokkaido University Hospital, Sapporo, Japan, hokudai.ac.jp; ^9^ Center for Genomic Medicine, Tonan Hospital, Sapporo, Japan, tonan.gr.jp

**Keywords:** cancer genomic profiling, genotype-matched therapy, gynecologic cancer

## Abstract

**Background and Aims:**

Personalized medicine utilizing comprehensive cancer genomic profiling (CGP) for gynecologic cancers is still in its early stages and faces numerous challenges. In this study, we aimed to elucidate the current status of CGP for gynecologic cancers at our institution.

**Methods:**

We prospectively analyzed 44 cases of gynecologic cancers that underwent CGP (FoundationOne CDx or Liquid CDx) from March 2020 to July 2022, evaluating the CGP results and clinical outcomes.

**Results:**

Forty‐one cases underwent FoundationOne CDx and four underwent FoundationOne Liquid CDx testing. The distribution of cancer types consisted of 10 cases of cervical cancer, 21 cases of ovarian cancer, 7 cases of endometrial cancer, and 6 cases of sarcomas. Actionable genomic alterations were identified in 42 cases (95.5%), with 16 cases (36.4%) presenting clinically for genotype‐matched therapy (GMT). However, GMT was administered in only three cases (6.8%). Among the cases without GMT, four experienced a deterioration in overall physical condition and two had complications as the reason for nonimplementation. Consideration of presumed germline pathogenic variants occurred in 10 cases (22.2%), with confirmatory testing conducted in two. In survival analysis using the Cox proportional hazards model, the presence of *PIK3CA* mutations was identified as being potentially associated with adverse prognosis (hazard ratio: 2.73, 95% confidence interval: 1.15–6.49, and *p* = 0.023).

**Conclusion:**

To enhance the prognosis of gynecologic cases, earlier CGP testing to expand the opportunities for GMT and the proactive introduction of *PIK3CA*‐related clinical trials might be crucial.

## 1. Introduction

Genotype‐matched therapy (GMT) for gynecologic cancers is rapidly becoming more widespread, driven by the emergence of companion diagnostic tests for detecting germline *BRCA1/2* gene mutations and homologous recombination deficiency–positive ovarian cancers, leading to the use of PARP inhibitors as GMT [1–4]. In Japan, treatment with the immune checkpoint inhibitor pembrolizumab for microsatellite instability‐high or mismatch repair deficiency solid tumors, primarily in endometrial cancer, is now included in public health insurance coverage [5–7].

Recently, comprehensive cancer genomic profiling (CGP) has become feasible, enabling trials to be conducted based on the Center for Cancer Genomics and Advanced Therapeutics (C‐CAT) framework (https://www.ncc.go.jp/jp/c_cat/index.html), even after the completion of standard systemic treatment covered by public health insurance. However, in the field of Japanese gynecologic oncology in particular, recommendations regarding the implementation of CGP have not been explicitly established. Although individual guidelines for each gynecologic cancer type (cervical cancer, endometrial cancer, and ovarian cancer, among others) have been published, only the latest edition of the cervical cancer guidelines (2022) explicitly mentions CGP, stating that “Cancer genomic profiling test at appropriate timings is suggested as well as treatments based on genomic variants” [8]. However, in gynecologic cancers other than cervical cancer, there are no clinical guidelines regarding CGP in Japan.

Therefore, the approach to CGP in clinical practice varies depending on the facility and attending physician and it has not yet been firmly established as standard treatment in the gynecology field in Japan. Meanwhile, numerous reports have explicitly stated the utility of CGP and recommended its clinical use [9–15]. Specialized reports in the field of gynecology also suggest that there are no clinical reasons to hesitate performing CGP [16, 17]. Nevertheless, the most significant barrier to the widespread adoption of CGP remains its cost [18]. CGP involves high testing costs due to the use of next‐generation sequencers for analysis, and the drugs administered based on the CGP results are molecular targeted therapies, imposing a substantial economic burden on treatment. Given the present circumstances of public health insurance in Japan, conducting CGP testing for cancer diagnosis or performing multiple CGP tests is not economically acceptable [19]. Therefore, it is even more crucial to identify the appropriate patients and correct timing for CGP testing.

Since CGP became eligible for public health insurance coverage in Japan in 2019, we have actively performed CGP for gynecologic cancers. The aim of this study was to investigate the utility of CGP and issues with its use in gynecologic cancers.

## 2. Methods

### 2.1. Patients and Study Design

**FIGURE 1:**
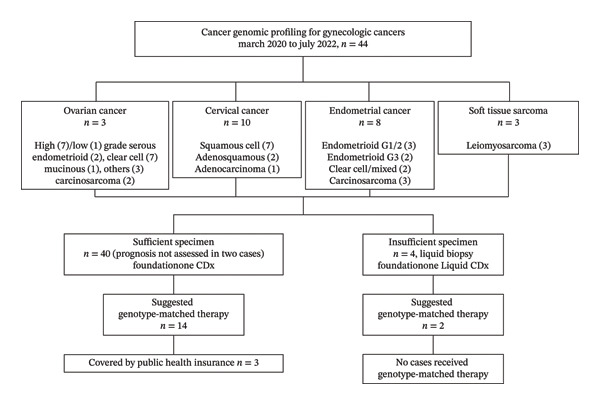
Case recruitment flow and implemented CGP methods. The flowchart shows the breakdown of the 44 cases of gynecologic cancer examined in this study, along with the types of CGP conducted and the status of GMT implementation.

This study was a prospective, single‐center, observational study of CGP in patients with gynecologic cancers conducted at Sapporo Medical University from March 2020 to July 2022 (Figure 1). The reporting of this study follows the STROBE guidelines to ensure standardized and transparent reporting of the study design and outcomes. Initially, 45 patients were identified as potential candidates for CGP; however, one patient declined to participate. Consequently, 44 patients who provided written informed consent were prospectively enrolled at the time CGP testing was first considered as a clinical option. The study protocol was reviewed and approved by the Institutional Ethics Review Board of Sapporo Medical University Hospital (approval number: 312‐64). The study was conducted in strict accordance with the ethical principles of the Declaration of Helsinki and its later amendments. Written informed consent was obtained from all individual participants prior to their inclusion in the study. Following enrollment, clinical data, including overall survival (OS) and treatment responses, were systematically tracked and updated in real time through medical records and patient interviews until the end of December 2022. This prospective approach provided significant methodological advantages by ensuring high data completeness and accuracy, thereby minimizing the recall bias and missing information often associated with retrospective designs. Due to the single‐center nature of the study, a formal power analysis for sample size was not conducted, and all analyses were performed as an exploratory analysis.

### 2.2. Genomic Analysis

**FIGURE 2:**
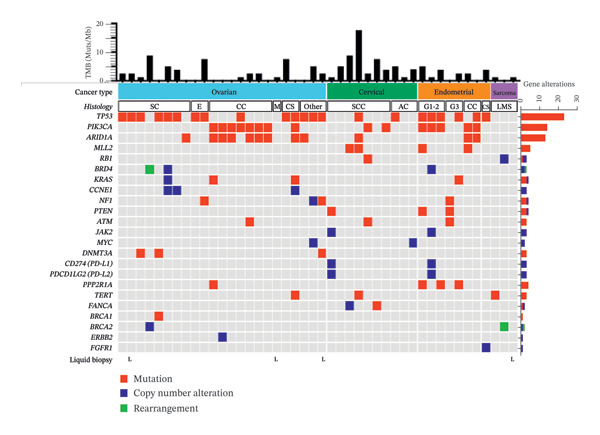
OncoPrint representation of genomic alterations. The OncoPrint diagram shows the detected genomic alterations in each case. The genes shown in this diagram either exhibited genomic alterations in three or more samples or were associated with GMT. The samples marked with “L” in the bottom row underwent CGP through liquid biopsy. AC: adenocarcinoma, CC: clear cell carcinoma, CS: carcinosarcoma E: endometrioid carcinoma, G1/2: endometrioid carcinoma grade 1 or 2, G3: endometrioid carcinoma grade 3, LMS: leiomyosarcoma, M: mucinous carcinoma, SC: serous carcinoma, SCC: squamous cell carcinoma.

The genes for which genomic alterations were detected in three or more cases among the actionable genomic alterations identified by F1 or F1L, or the genes considered as targets for GMT, are presented in an OncoPrint format (Figure 2). GMT recommendations were determined through a consensus process by the multi‐institutional Molecular Tumor Board (MTB) based at Sapporo Medical University Hospital and Hokkaido University Hospital. The MTB reviewed the F1/F1L reports to identify actionable variants and assigned evidence levels according to the Clinical Practice Guidance for Next‐Generation Sequencing in Japan [20, 21]. The feasibility of GMT was then evaluated based on the specific genomic findings, the availability of corresponding clinical trials or approved drugs, and the patient’s individual clinical condition.

### 2.3. Responses to GMT

The OS rate was defined using the Response Evaluation Criteria in Solid Tumors Version 1.1 as assessed by the investigators [22].

### 2.4. Statistical Analysis

Statistical analysis was performed in accordance with the SAMPL guidelines and the recommendations by Assel et al. [23]. Given the single‐institution nature and the sample size of this study, all statistical analyses were considered exploratory. Analysis was conducted using IBM SPSS Statistics for Windows, Version 27.0 (IBM Corp., Armonk, NY, USA). OS was calculated from the date of gynecological cancer diagnosis and treatment initiation until death from any cause or the last follow‐up at the end of December 2022. Survival curves were estimated using the Kaplan–Meier method, and the log‐rank test was used to evaluate differences between groups. The Cox proportional hazards model was employed for multivariable analysis to estimate hazard ratios and identify potential prognostic associations. Variables for the model were selected using a stepwise method with a *p* value threshold of < 0.10. Given the small sample size (*n* = 44), all multivariable analyses should be considered exploratory, and the results are presented as potential associations rather than definitive independent predictors to account for the risk of overfitting and model instability. All statistical tests were two sided, and an a priori significance level was set at *p* < 0.05.

### 2.5. Evaluation of Presumed Germline Pathogenic Variants and Genetic Counseling

Evaluation of presumed germline pathogenic variants and genetic counseling: According to the recommendations of the AMED Kosugi group (supported by the Japan Agency for Medical Research and Development) [24], certified genetic counselors and clinical geneticists assessed the presumed germline pathogenic variants. Subsequently, whether presumed germline pathogenic variants should be disclosed or not was decided by the MTB.

## 3. Results

### 3.1. Patient Characteristics and CGP

The demographic characteristics of the 44 cases examined in this study are summarized in Table 1 and Figure 1. The distribution of cancer types consisted of 10 cases of cervical cancer, 23 cases of ovarian cancer (including 2 carcinosarcomas), 8 cases of endometrial cancer (including 1 carcinosarcoma), and 3 cases of soft tissue sarcoma (all leiomyosarcomas). The majority (75.0%) of the cases had advanced cancer, at Stage III or higher, at the initial diagnosis. The most common types were high‐grade serous carcinoma and clear cell carcinoma of ovarian cancer, as well as squamous cell carcinoma of cervical cancer, with seven cases of each type. Among the total cases, 31 (70.5%) had already received three or more chemotherapy regimens. The majority of specimens used for CGP were surgical specimens (63.6%) with an adequate tumor volume; liquid biopsy was performed in four cases (9.1%) because of a low tumor volume.

**TABLE 1:** Patient characteristics.

	***n* (%)**

Age, years	53.8 (28–75)
ECOG performance status	
0	35 (79.5)
1	9 (20.5)
FIGO staging	
I	10 (22.7)
II	1 (2.3)
III	22 (50)
IV	11 (25)
Cancer type and histology	
Ovarian cancer	
High‐grade serous	7 (15.9)
Low‐grade serous	1 (2.3)
Endometrioid	2 (4.5)
Clear cell	7 (15.9)
Mucinous	1 (2.3)
Ovarian carcinosarcoma	2 (4.5)
Mixed‐type adenocarcinoma	1 (2.3)
Primary peritoneal cancer	1 (2.3)
Malignant Brenner tumor	1 (2.3)
Cervical cancer	
Squamous cell	7 (15.9)
Adenosquamous	2 (4.5)
Adenocarcinoma	1 (2.3)
Endometrial cancer	
Endometrioid G1/G2	3 (6.8)
Endometrioid G3	2 (4.5)
Clear cell	1 (2.3)
Mixed‐type endometrioid with clear cell	1 (2.3)
Uterine carcinosarcoma	1 (2.3)
Sarcoma	
Leiomyosarcoma	3 (6.8)
Previous lines for therapy	
1	3 (6.8)
2	10 (22.7)
3	12 (27.3)
> 3	19 (43.2)
Sample collection	
Operation (primary site)	23 (52.3)
Operation (metastatic site)	5 (11.4)
Biopsy (primary site)	8 (18.2)
Biopsy (metastatic site)	4 (9.1)
Liquid biopsy (blood)	4 (9.1)

### 3.2. Genomic Landscape of Gynecologic Cancers

Figure 2 presents an OncoPrint of the actionable genomic alterations detected by CGP. Actionable genomic alterations were identified in 42/44 cases (95.5%), and the most frequently observed genomic alteration across all gynecologic cancers was in *TP53* (22 cases, 50.0%), followed by *PIK3CA* (15 cases, 34.1%) and *ARID1A* (12 cases, 27.3%), all of which were mutations (single nucleotide variation or short indel). Other genomic alterations were observed in only a few cases for each gene, and in addition to mutations, copy number alterations and genomic rearrangements were identified, but no specific tendencies were observed. A detailed analysis of the 15 cases with *PIK3CA* alterations revealed that they were most prevalent in specific histological subtypes: all 7 cases of ovarian clear cell carcinoma (OCCC) (100%), 3 of the 10 cases of cervical cancer (all squamous cell carcinoma), and 5 of the 8 cases of endometrial cancer (3 endometrioid G1‐2 and 2 clear cell adenocarcinoma). Regarding tumor mutation burden, only one case of cervical cancer was classified as tumor mutation burden high (17.65 mutations/Mb).

In all cases, presumed germline pathogenic variants were discussed by the MTB with a clinical genetics expert. Genetic counseling was provided to the 10 cases with an identified presumed germline pathogenic variant, but only two cases requested germline testing. The detection of pathogenic variants was confirmed in only one case of ovarian cancer with a germline variant in *PALB2* (Table S1).

### 3.3. Targeted Genetic Alterations and GMT

Table 2 presents details of the cases who were recommended to receive GMT according to the results of F1 and F1L testing. The most common cancer types for which GMT was offered were ovarian cancer (8 cases in F1, 2 cases in F1L cases). Finally, three cases received GMT. Patient 4 and Patient 6, both with ovarian cancer harboring *ATM* and *BRCA1* mutations, respectively, received PARP inhibitors. Patient 9, who had TMB‐high cervical cancer, was treated with pembrolizumab. Among them, two cases of ovarian cancer were administered a PARP inhibitor (olaparib or niraparib), and one tumor mutation burden‐high case underwent treatment with pembrolizumab as part of public health insurance‐covered treatments.

**TABLE 2:** Summary of GMT for the result of CGP.

Pt. No.	Cancer type	CGP test	Targeted genetic alteration	Previous lines	Underwent GMT	Reason for not implementing GMT
1	Ovarian cancer	F1	LOH score (HRD positive), NF1 mutation	15	NA	Deterioration in overall physical condition
2	Ovarian cancer	F1	PIK3CA mutation	2	NA	Deterioration in overall physical condition
3	Ovarian cancer	F1	KRAS mutation	4	NA	Ineligible for the clinical trial (thrombosis)
4	Ovarian cancer	F1	*ATM mutation*	1	PARP inhibitor	—
5	Ovarian cancer	F1	TP53 mutation	3	NA	Wanted to continue standard treatment
6	Ovarian cancer	F1	*BRCA1* mutation	1	PARP inhibitor	—
7	Ovarian cancer	F1	TP53 mutation	6	NA	Wanted to continue standard treatment
8	Ovarian cancer	F1	ERBB2 amplification	1	NA	Wanted to continue standard treatment
9	Cervical cancer	F1	Tumor mutation burden (TMB) high	2	Immune checkpoint inhibitor	—
10	Cervical cancer	F1	FANCA loss	3	NA	Deterioration in overall physical condition
11	Endometrial cancer	F1	PD‐L1/PD‐L2 amplification	2	NA	Deterioration in overall physical condition
12	Endometrial cancer	F1	TP53 mutation	2	NA	Wanted to continue standard treatment
13	Uterine cacinosarcoma	F1	FGFR1 amplification	3	NA	Distant location of the clinical trial facility
14	Ovarian carcinosarcoma	F1	PIK3CA mutation	3	NA	Ineligible for the clinical trial (severe anemia)
15	Ovarian cancer	F1L	TP53 mutation	4	NA	Needed additional biopsy for trial entry
16	Ovarian cancer	F1L	NF1 mutation	9	NA	Wanted to continue standard treatment

*Note:* F1, FoundationOne CDx genome profiling; F1L, FoundationOneLiquid CDx genome profiling.

Abbreviations: CGP, cancer genomic profiling; GMT, genotype‐matched therapy; NA, not assessed.

Regarding the 13 cases who were recommended GMT but did not receive it, the primary reason was the timing of the intervention. Six cases (46.2%) were unable to initiate therapy due to rapid clinical deterioration (*n* = 4) or complications (*n* = 2) shortly after the CGP results were available. Of the remaining cases, five continued to receive standard treatment, one faced geographical barriers to the trial site, and one required an additional biopsy that was clinically unfeasible. These results underscore the “real‐world” challenge in Japan where CGP is often performed after exhausting standard lines, frequently leaving an insufficient window for GMT.

### 3.4. Genomic Alterations and Clinical Prognosis

Table 3 presents the results of our analysis on OS. We examined each clinical factor and frequent genomic alterations using Cox proportional hazards regression analysis, and *PIK3CA* alteration was identified as a factor potentially associated with poor prognosis (hazard ratio: 2.73, 95% confidence interval: 1.15–6.49, and *p* = 0.023). The clinical background of these 15 *PIK3CA*‐mutated cases confirmed a late‐stage, heavily pretreated status; 80.0% (12/15) had undergone three or more previous chemotherapy regimens prior to CGP testing. Despite having a relatively preserved performance status (ECOG PS 0 or 1) at the time of enrollment, these patients exhibited rapid progression compared with the *PIK3CA* wild‐type group, with a significant correlation with worse OS observed in both the total cohort (*p* = 0.046) and the ovarian cancer subgroup (*p* = 0.011) (Figure S1).

**TABLE 3:** Analyses of clinical and genomic features with overall survival using the cox proportional hazards model.

Factor	Overall survival univariate HR (95% CI)	*p*	Overall survival multivariate HR (95% CI)	*p*
Age	0.98 (0.95–1.00)	0.087	0.97 (0.94–1.00)	0.061
ECOG performance status				
0	1	0.87		
1	1.08 (0.43–2.70)			
Cancer type				
Ovarian cancer	1	0.54		
Cervical cancer	1.81 (0.71–4.65)	0.22		
Endometrial cancer	1.80 (0.62–5.21)	0.28		
Sarcoma	1.07 (0.30–3.83)	0.92		
FIGO staging				
I/II	1	0.13	1	0.062
III/IV	2.15 (0.81–5.72)		2.61 (0.95–7.15)	
Genomic alteration				
TP53				
−	1	0.73		
+	0.87 (0.41–1.87)			
PIK3CA				
−	1	**0.025**	1	**0.023**
+	**2.56 (1.12–5.85)**		**2.73 (1.15–6.49)**	
ARID1A				
−	1	0.14		
+	1.89 (0.82–4.37)			
Tumor mutation burden	0.95 (0.85–1.06)	0.39		
Received GMT				
No	1	0.30		
Yes	0.34 (0.05–2.55)			

Abbreviations: CI, confidence interval; GMT, genotype‐matched therapy; HR, hazard ratio.

*Note:* Bold values indicate statistical significance at *p* < 0.05.

## 4. Discussion

In the present study, we prospectively followed the implementation of comprehensive CGP for gynecologic cancers at a single institution and identified several critical issues in the real‐world clinical landscape of Japan. Notably, we quantified a significant “timing gap”: 46.2% of the candidates for GMT lost the opportunity to receive treatment due to rapid deterioration in their overall physical condition while waiting for CGP results (Table 2). Furthermore, our exploratory analysis suggested that the presence of *PIK3CA* alterations is potentially associated with poor prognosis in heavily pretreated gynecologic cancers (HR: 2.73 and *p* = 0.023) (Table 3). These findings, derived from a prospective design that ensured high data completeness, highlight the urgent clinical necessity of reconsidering the timing of CGP to maximize the therapeutic window for GMT.

The primary reason that CGP was performed at a late stage in our cohort—where 70.5% of the patients had already received three or more chemotherapy regimens—is the specific reimbursement criteria under the Japanese public health insurance system [19, 21]. Currently, public insurance in Japan covers CGP only for patients with solid tumors who have completed or are expected to complete standard systemic therapies. While our study demonstrates that this “standard treatment after” approach often results in “too late” intervention, the timing in this study strictly adhered to these national regulations. Therefore, our results underscore a fundamental conflict between institutional rules and clinical needs. This “timing gap” issue is the subject of active investigation; for instance, the UPFRONT trial (NCCH1908), which evaluates the utility of CGP before the initiation of first‐line systemic therapy, is currently ongoing in Japan [25]. Our findings regarding the high rate of clinical deterioration support the premise of such trials and underscore the need for earlier genomic intervention.

Regarding the prognostic impact of *PIK3CA* alterations, our study expands on previous histological reports by suggesting that it is a factor potentially associated with poor prognosis across advanced gynecologic cancers, even after adjusting for the FIGO stage (Table 3). This aligns with the high frequency of *PIK3CA* alterations in Japanese OCCC (57%) and endometrial cancers (40%) in the national C‐CAT database [26]. Crucially, the therapeutic landscape for these high‐risk patients has recently shifted. In March 2026, risovalisib (CYH33), a novel selective PI3Kα inhibitor, was approved in Japan for patients with *PIK3CA*‐mutated OCCC [27, 28]. This milestone was based on the results of the international Phase II G201 trial, which reported a significant objective response rate (ORR) of 34.5% [28, 29]. Although a single‐gene companion diagnostic (CDx) for risovalisib is now available, the early implementation of CGP remains the superior clinical strategy [21]. CGP simultaneously evaluates multiple other potential targets and clinical trial eligibility in a single intervention [20]. Given that public insurance in Japan covers CGP only once per lifetime, utilizing this single opportunity early to establish a comprehensive “genomic roadmap” is essential to ensure that patients do not miss the window of opportunity for GMT, including newly approved agents like risovalisib.

Following the Trial of Onco‐Panel for Gene‐profiling to Estimate both Adverse events and Response (TOP‐GEAR) project in Japan, several CGP platforms became available since 2019 [14, 20]. While these platforms have expanded access, drug accessibility remains a challenge, as evidenced by our low GMT implementation rate (6.8%) [19]. Beyond *PIK3CA*, our study identified high prevalence of *TP53* (50.0%) and *ARID1A* (27.3%) alteration. Although these did not reach statistical significance in association with prognosis in our cohort, their clinical relevance remains substantial. *TP53* mutations were predominantly observed in aggressive subtypes like high‐grade serous ovarian cancer and squamous cell carcinoma of the cervix, consistent with their role as foundational drivers of genomic instability. Similarly, *ARID1A* mutations were frequent in OCCC and endometrioid carcinomas, often co‐occurring with *PIK3CA* alterations [26]. This synergistic genomic signature is closely associated with resistance to conventional platinum‐based chemotherapy in the Japanese population [26, 30]. Future clinical trials exploring synthetic lethality or targeted approaches for these frequent mutations are needed to overcome the intractable nature of these cancers.

This study has several limitations that must be considered. First, as a prospective, single‐center study with a relatively small sample size (*n* = 44), the findings should be regarded as exploratory and may not be fully generalizable to the broader population. In particular, the multivariable results regarding *PIK3CA* should be interpreted with caution due to the risk of overfitting and model instability inherent in small datasets using a stepwise variable selection approach. Second, our cohort was heavily weighted toward ovarian cancer, particularly clear cell adenocarcinoma, which may introduce histological bias. Third, the Japanese insurance system’s “once‐per‐lifetime” restriction inherently influences testing timing and may create a selection bias toward patients who have already exhausted standard treatments. Finally, the low GMT implementation rate reflects real‐world challenges in drug accessibility and the rapid clinical deterioration of late‐stage patients.

In summary, it is crucial to propose GMT before clinical deterioration. Reconsidering the timing of the one‐time CGP to be earlier is essential to maximize the clinical benefit of emerging therapies in Japanese gynecologic oncology.

## Author Contributions

Tasuku Mariya, Kohichi Takada, and Akihiro Sakurai: conceptualization and methodology. Kazusa Nofuji, Tasuku Mariya, Shutaro Habata, and Masashi Idogawa: visualization, investigation, software, and writing–original draft preparation. Tomohiro Kubo, Ayako Murota, Mizue Teramoto, Motoki Matsuura, Masahiro Iwasaki, and Aki Ishikawa: data collection and analysis. Ichiro Kinoshita and Tsuyoshi Saito: supervision.

Tasuku Mariya had full access to all of the data in this study and takes complete responsibility for the integrity of the data and the accuracy of the data analysis. Tasuku Mariya affirms that this manuscript is an honest, accurate, and transparent account of the study being reported; that no important aspects of the study have been omitted; and that any discrepancies from the study as planned (and, if relevant, registered) have been explained.

## Funding

This work was supported by JSPS KAKENHI, Grant numbers JP20K16363, JP23K08849, and JP24K12603.

## Disclosure

All authors have read and approved the final version of the manuscript.

## Conflicts of Interest

Koichi Takada received lecture fee from Daiichi Sankyo Co. Ltd., Chugai Pharmaceutical Co. Ltd., Eisai Co. Ltd. and Sysmex corporation. All the other authors declare no conflicts of interest.

## Supporting Information

Additional supporting information can be found online in the Supporting Information section.

## Supporting information


**Supporting Information 1** Supporting Figure 1. Kaplan–Meier survival curves comparing overall survival between patients with and without *PIK3CA* alterations in the total cohort and the ovarian cancer subgroup.

**Supporting Information 2** Supporting Table 1. Summary of presumed germline pathogenic variants identified by CGP, including the status of genetic counseling and germline confirmatory testing for each case.

## Data Availability

The data that support the findings of this study are available from the corresponding author upon reasonable request.
