# Supplementary figures and images for: Earlier Comprehensive Cancer Genomic Profiling in Gynecologic Cancers May Facilitate Genotype‐Matched Therapy: A Prospective Single‐Institution Study
Source: Obstet Gynecol Int. 2026 Jun 18;2026:9990671. doi: 10.1155/ogi/9990671 (PMC13277764; doi:10.1155/ogi/9990671)

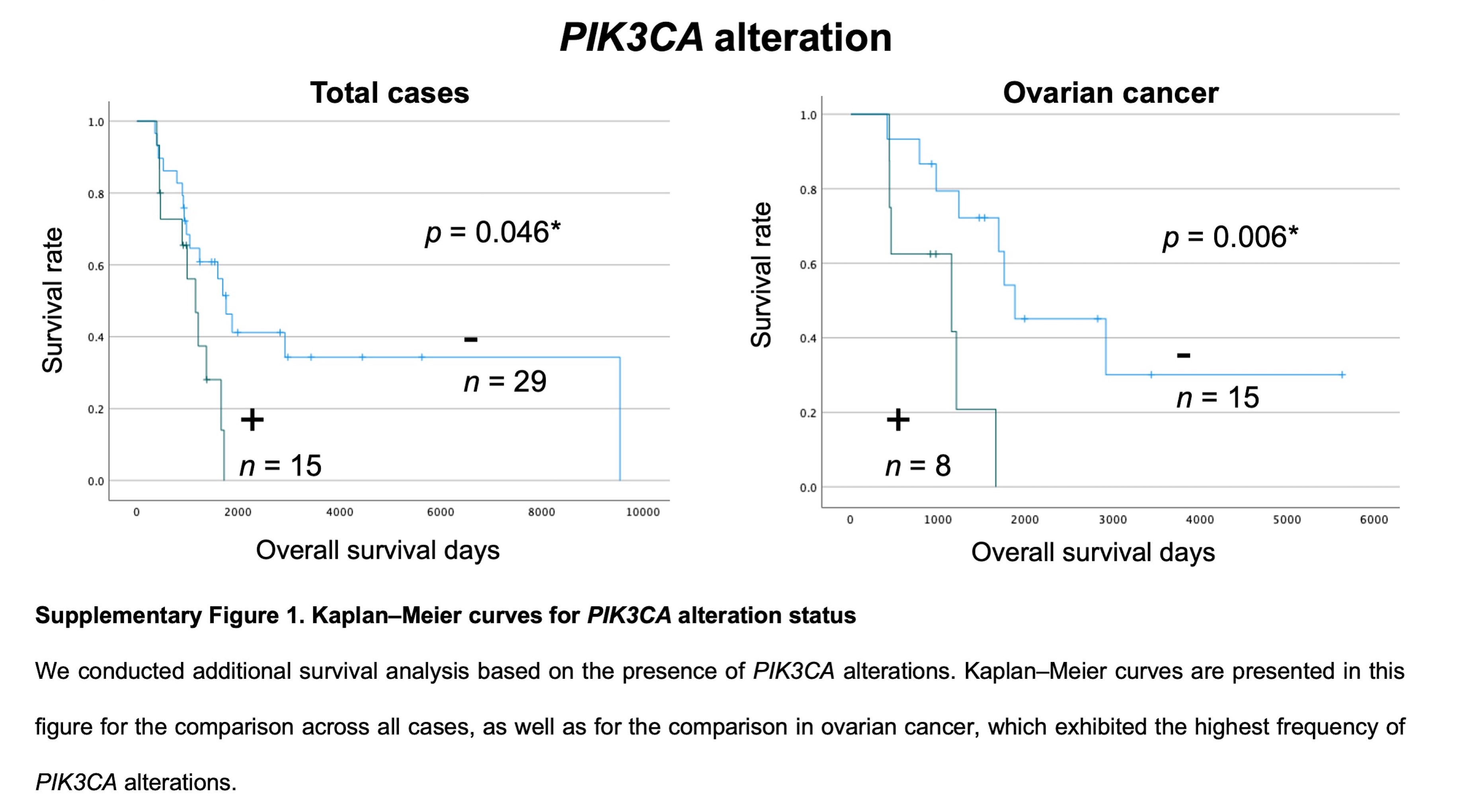

Supplement: Supplementary file 1 — Supporting Information 1 Supporting Figure 1. Kaplan–Meier survival curves comparing overall survival between patients with and without PIK3CA alterations in the total cohort and the ovarian cancer subgroup. [file OGI-2026-9990671-s002.jpeg]
